# Collis' Method of Operating for Hare-Lip

**Published:** 1873-09

**Authors:** Theodore A. McGraw

**Affiliations:** Professor of Surgery in Detroit Medical College.


### ARTICLE VI.

### *Collis' Method of Operating for Hare-lip.*

By Theodore A. McGraw, M. D.

Professor of Surgery in Detroit Medical College.

It is to me a very singular fact that the operation for hare-lip, invented by Mr. Collis, of Dublin, a few years ago, is not described in any of our text-books on surgery. It certainly is superior in its results to any of the old methods, and it was with great disappointment that I found, on reviewing Prof. Hamilton's work on surgery, that he still recommends a mode of operating which is not only out of date, but also exceedingly faulty. If we operate on a patient according to the method described in Prof. Hamilton's work, and union takes place without suppuration, it will seem, at first, to be a success. This success is, however, only apparent, and speedily gives way to a deformity, caused by the contraction of the cicatrix. Where the cut edges were united the new lip will be very thin, and its border will be indented. To obviate this indentation of the lip, and to give it a normal thickness, I am accustomed to resort to the procedure of Dr. Collis, of Dublin. This method is not as well understood by the profession as its merits deserve, and I will therefore briefly describe it.

The essential feature of the operation is the insertion of a flap taken from one side of the cleft into the other side, which is split to receive it. A flap is cut in the usual manner from one side of the cleft, involving the whole thickness of the lip. This flap is left attached to the vermilion border of the lip. The lip on the other side of the cleft is simply split open from the upper edge of the cleft nearly to the corner of the mouth. By inserting the flap into this split we will evidently increase the thickness of the lip. The resulting scar will be not perpendicular, but longitudinal, and its contraction would make the lip smaller in size, but could not possibly cause any irregularity of its border.—*Detroit Review of Medicine.*

---

